# Unravelling the role of electrocardiogram changes and ejection fraction in ischaemic stroke outcomes

**DOI:** 10.1186/s43044-025-00624-4

**Published:** 2025-06-04

**Authors:** Anna Meijia Jiang, Lechkna Paras Chajed, Maham Munir Malik, Janani Lambotharan, Josh Williams, Jack Plume, Brian Wang

**Affiliations:** 1 St George’s Hospital Medical School, London, SW17 0RE UK; 2 UCL Medical School, University College of London, London, WC1E 6BT UK; 3 Faculty of Biology, Medicine and Health, University of Manchester, Manchester, M13 9PL UK; 4 Leicester Medical School, University of Leicester, Leicester, UK; 5 Faculty of Life Sciences & Medicine, King’s College London, London, UK; 6 Jersey Heart Team, The General Hospital, Gloucester Street, St Helier, Jersey

**Keywords:** ECG, Ejection fraction, Stroke, Stroke-heart

## Abstract

**Background:**

Stroke, a prevailing global cause of mortality, is witnessing a surge in incidences, particularly in lower-income countries. However, existing guidelines fail to adequately address the impact of electrocardiogram (ECG) changes and ejection fraction on the outcomes of ischaemic stroke, as well as the management of stroke-heart syndrome.

**Main Body:**

Existing literature underscores a strong link between ischaemic stroke and subsequent cardiac manifestations, hinting at their potential as prognostic indicators for adverse stroke outcomes. Post-ischaemic stroke ECG changes correlate with heightened heart disease risks, emphasising the need for post-stroke ECG monitoring. Recommendations include the use of direct oral anticoagulants and warfarin within 14 days of stroke for atrial fibrillation and thrombolytics for other ischaemic strokes. Conflicting findings exist on the prognostic significance of lower left ventricular ejection fraction (LVEF) post-ischaemic stroke, with some studies indicating poorer outcomes. Currently, LVEF does not significantly impact managing ischaemic stroke patients, but anticoagulation may be considered. Stroke-heart syndrome, a rare post-stroke complication, lacks clear understanding and guidelines for physicians.

**Conclusions:**

ECG changes emerge as potential stroke outcome predictors, with ongoing debate on the utility of lower LVEF. While an ABC approach shows some efficacy for stroke-heart syndrome, additional research is crucial to unravel its ambiguous physiology and clarify these uncertainties.

## Background

In 2018, stroke ranked as the fourth leading cause of death in the UK, contributing to 75% of all cerebrovascular disease-related fatalities, with a declining trend in annual rates [1]. However, the global landscape in 2021 witnessed a worrisome surge in stroke incidence by 70%, stroke prevalence by 2%, and stroke-related disability-adjusted life years (DALYs) by 43% [2]. This alarming escalation disproportionately affects lower and lower-middle-income countries, where an estimated 86% of stroke deaths occur, highlighting significant geographical disparities [2]. Ischaemic stroke, constituting 62% of all strokes, strikes individuals under 70 years old in 58% of cases [2]. Despite atrial fibrillation (AF) and atrial flutter being acknowledged risk factors, impacting 25% of stroke patients, these conditions are surprisingly excluded from the Global Burden of Disease (GBD) for stroke [2, 3]. This omission underscores the interconnectedness of ischaemic stroke with cardiovascular factors, revealing a critical gap in addressing these risk factors within stroke management.

Stroke-induced cardiac alterations manifest diversely, and electrocardiogram (ECG) changes serve as potential indicators of stroke [4]. While only 7.6% of ischaemic stroke patients exhibit clinically relevant ECG findings, the presence of ST depression and Q waves can signal acute ischaemic stroke [5, 6]. These ECG changes correlate with increased troponin T, indicating cardiac involvement and foreshadowing unfavourable outcomes [6].

The intricate physiological link between the heart and brain implies that malfunction or failure in one organ can adversely affect the other. Heart failure not only predisposes patients to stroke through various mechanisms, including cardioembolism, but also exacerbates post-stroke outcomes [4, 7]. This well-established connection is underscored by studies focussing on preventing ischaemic stroke secondary to heart failure, primarily involving anticoagulants [8]. Additionally, the shared risk factors between stroke and cardiovascular disease underscore their bidirectional relationship [9]. Conversely, ischaemic stroke induces anoxic damage to the brain parenchyma, triggering organ dysfunction beyond the central nervous system, and affecting organs such as the kidneys, liver, and lungs [10].

The aftermath of ischaemic stroke, termed stroke-heart syndrome, carries clinical significance as cardiac events substantially contribute to post-stroke morbidity and mortality. Cardiac causes account for 2–6% of post-stroke deaths [11–13], with approximately 9% of ischaemic stroke patients experiencing subsequent cardiac events [14]. Recognising stroke-heart syndrome globally as a major contributor to death and disability, increased awareness and diagnosis among clinicians could enhance outcomes for patients, reinforcing the bidirectional nature of the stroke-heart relationship.

This review aims to explore the potential link between ECG changes post-ischaemic stroke and assess their predictive value for patient outcomes. Updated guidelines regarding the influence of different ECG changes on anticoagulant medication use will be examined. Furthermore, the review will delve into the cardiac parameter of ejection fraction concerning stroke, along with an exploration of the current understanding of the pathophysiology and management of stroke-heart syndrome.

## Main text

Continuous ECG monitoring in post-stroke patients holds significant importance owing to its predictive nature, contributing to enhanced mortality rates and the evaluation of cardiac damage extent [15]. Manea et al. underscored the closely intertwined relationship between cardiac arrhythmias and stroke [15]. Notably, atrial fibrillation (AF), a prevalent form of cardiac arrhythmia, stands as one of the most common aetiologies of acute strokes [15]. Current guidelines are that monitoring post-stroke for 24 h is important in  the identification of AF, and further monitoring of ≥ 72 h can be done in patients who have had an ischaemic stroke or transient ischaemic attacks [16]. However, these monitoring guidelines are more aimed at hospital admissions. Community-based care is often not included in guidelines, but Lyckhage et al. conducted a study utilising GP lead ECG monitoring in Denmark [17]. Patients without previous AF and who had an ischaemic stroke were followed up for 1 year after, using a 7-day Holter ECG, and  4.6% of these patients had new-onset AF [17]. Conversely, cardiac arrhythmias rank as the primary cause of mortality in acute stroke patients [15]. Stroke-induced cardiac changes manifest independently of pre-existing cardiovascular disease, with up to one-third of post-stroke patients exhibiting ECG changes having no prior cardiovascular conditions [15, 18]. However, these changes are contingent upon the stroke type and location, with haemorrhagic stroke patients being 1.5 to 2.3 times more likely to develop ECG changes than their ischaemic stroke counterparts [15]. Consequently, ECG monitoring in post-stroke patients facilitates early detection and correction of cardiac arrhythmias, potentially averting sudden death if left unnoticed.

### Prevalence of ECG changes

The utilisation of ECG monitoring serves as a valuable tool in determining myocardial injury inflicted by strokes and assessing its severity by identifying indicative signs of ischaemic damage. In a clinical observational study conducted by Zhang et al., involving 200 acute stroke patients over a two-year period, ECG changes were closely monitored post-stroke [19]. Their findings revealed that 72% of these patients exhibited an ischaemic ST segment change, while 9.5% experienced arrhythmias following the stroke [19]. Notably, the severity of the stroke was assessed using the specific finding of ischaemic ST segment changes [19]. The efficacy of ECG monitoring within the initial 24 to 48 h post-stroke was underscored, as pathological findings during this period were reversible, leading to improved prognosis when early detection occurred [19]. However, it is important to note that the study's reliance on a small sample size of 200 patients warrants further investigation with a larger cohort for confirmation.

Supporting the critical timing of ECG monitoring, Kallmünzer et al. conducted a prospective observational study highlighting the significance of monitoring within the first 24 h after stroke admission [20]. Among the 501 patients monitored, 74% exhibited signs of cardiac arrhythmias within the initial 24 h, indicating this period as the highest risk for incidents to occur [20]. While the greatest number of serious cardiac consequences, such as cardiac death and myocardial infarction, occurred during days 2 and 3 post-stroke, the prevalence of arrhythmias decreased between the first 24 h and day 3 [20]. This emphasises that ECG monitoring for arrhythmias is most crucial during the initial 24 h post-stroke, and despite a decline in prevalence, serious cardiac events could still transpire up to 3 days later.

Numerous ECG changes have been identified in post-ischaemic stroke patients, and theorised as predictive factors for subsequent ischaemic strokes [15]. These changes encompass ST segment abnormalities, negative T-waves, U waves, left axis deviation, prolonged QT interval, atrial fibrillation, atrial flutter, sinus tachycardia, ventricular tachycardia (VT), atrial and ventricular premature complexes, and bradyarrhythmias [15]. The location of stroke-induced damage, particularly in the insular cortex, appears to influence the presence of bradycardic or tachycardic arrhythmias [15]. Damage to the rostral posterior insular cortex is associated with tachyarrhythmias on ECG due to sympathetic nervous system stimulation, while bradyarrhythmias are more common following damage to the caudal posterior insular cortex, stimulating the parasympathetic nervous system [15]. The tachyarrhythmias were shown to exacerbate the extent of cerebral damage following the stroke. Among these arrhythmias, AF, atrial flutter, and VT are notably prevalent, with AF strongly linked to stroke and contributing to the risk of thromboembolism [21]. However, new-onset AF has a lower prevalence in ischaemic stroke patients, suggesting it might be a cause rather than a product of ischaemic stroke [18, 21].

Arrhythmias were also a common presentation in patients with cardioembolic strokes of the middle cerebral artery territory, with an increased risk associated with age and the National Institutes of Health Stroke Scale (NIHSS) score on admission [20, 22]. Hays et al. found a significantly higher NIHSS score of ≥ 6 in embolic stroke patients (66%) than any other subtypes (41.6%) [23].

The sympathetic nervous system relating to these arrhythmias was found to trigger a surge in catecholamines and cortisol, leading to tachycardia, which inflicts damage to the myocardium [15]. This damage is reversible; hence, employing a non-invasive technique like ECG monitoring post-stroke would enable early detection to prevent further complications [15]. In contrast to bradyarrhythmias and tachyarrhythmias, which are contingent on the site of damage within the insular cortex, systolic and diastolic dysfunction can be observed in any type of stroke [15]. These dysfunctions can also be monitored using ECG changes, in addition to monitoring cardiac markers in the blood, such as cardiac troponin [15].

Ibrahim et al. also underscored the significance of the affected stroke region in ECG changes [24]. Patients affected by the most common infarct, the temporoparietal lobe, predominantly exhibited tachycardias, while patients with occipital, occipital–parietal, and subarachnoid infarcts presented with bradycardias [24]. Additionally, Zhang et al. observed that patients with ischaemic stroke, encompassing lacunar, small, and large infarcts, often manifested ischaemic ST (14.1%) and T-wave (16.7%) changes in the ECG, with non-ischaemic patients being unlikely to present with ischaemic ECG changes [19, 22]. Specifically, for large ischaemic infarcts, bundle branch block and arrhythmias were likely to be present [22]. These ischaemic ECG changes are defined by Zhang et al. as ST-T changes of 0.5mv or more; T-waves changes of double hump, inversion or low-flat [19]. As ischaemic stroke can be caused by cerebral thrombosis or cerebral embolus, ECG changes differ concerning the cause [25]. Goldstein has found a significant 47% of cerebral emboli patients has AF at the time of the study, and no cerebral thrombotic patients were found with AF [26]. Unfortunately, in patients with acute ischaemic stroke and AF, Paciaroni et al. have found an increase in haemorrhagic transformation (11%) when compared to the average of 9% [27]. Those patients who had a haemorrhagic transformation were significantly more likely to experience recurrent ischaemic stroke, have higher mortality, and become disabled [27]. Although no direct evidence of the relationship between haemorrhagic transformation of ischaemic stroke and ECG changes has been found, it is known that haemorrhagic strokes do commonly present with “QT-c prolongation, ST segment abnormalities, and bradycardia or tachycardia” [28]. It may be possible to imply that if these changes are seen in ischaemic stroke patients, it could predict or confirm the transformation of a haemorrhagic stroke.

Despite all these findings of possible ECG changes associated with stroke, it can be difficult to distinguisg new from old when no ECGs are available for comparison. However, certain clues can help clinicians differentiate acute from chronic. Notably neurological trauma, including ischaemic stroke, expresses a specific ECG abnormality where T-waves are deep, diffused, and symmetrically inverted. These changes are called cerebral T-waves and can be used clinically to rule in ischaemic stroke, and they are mostly present in acute settings [29, 30]. Alternatively, Lancellotti et al. have found that echocardiograms can be used to establish chronic or acute cardiac changes, which can be utilised when determining whether the preserved ECG changes are new [31].

Furthermore, a cohort study by Olma et al. looked at ECG results of acute and transient ischaemic stroke patients 24 months after the stroke episode and found that 5.3% of patients had abnormal findings on ECG, and 14.8% of those patients were newly detected with AF [22]. However, this study did not specifically discuss ischaemic stroke patients and some patients received pacemakers or beta blockers during the follow-up. Therefore, any cardiac arrhythmias in those who received pacemakers will not be detectable and possibly generate a falsely low percentage of abnormal ECG findings in post-stroke patients.

The pathophysiology behind ECG changes post-ischaemic stroke remains incompletely understood, but a sympathetic theory has been discussed [32]. Myers et al. have shown that patients who had ischaemic stroke had significantly elevated levels of the sympathetic neurotransmitters, catecholamines, than those who did not [33]. These catecholamines have been found by Boukens et al. to stimulate b-adrenergic receptors, which can induce T-wave polarity, including T-wave inversion [34]. However, Boukens et al. did conduct this study using ball pythons and similar studies have yet to occur in humans. Other causes of ECG changes post-stroke may be related to electrolyte abnormalities [35]. It is reported that post-acute stroke, 18.7% of patients experience hypokalaemia, 10–20% have hypomagnesaemia, and 5,9% have hypophosphatemia [35]. Although the systematic review does not state any ECG changes were measured post-stroke, these electrolyte imbalances are known to be identifiable on ECG.

Despite the compelling evidence supporting the importance of ECG monitoring post-stroke, distinguishing whether cardiac arrhythmias are causative or consequential to the stroke remains challenging. Risk factors such as hyperlipidaemia, smoking, hypertension, and a history of transient ischaemic attack (TIA) may increase the likelihood of a stroke [24]. Ibrahim et al. emphasised that existing cardiac arrhythmias could serve as additional risk factors for stroke causation rather than being solely consequences of the stroke itself [24]. Therefore, determining if the stroke is the actual instigator of the cardiac arrhythmias detected through ECG monitoring is imperative [24] Table 1.

**Table 1:** Prevalence of ECG changes post-stroke in relavent studies

Electrocardiogram changes	Zhang et al. [19]	Kallmünzer et al. [20]	Olma et al. [22]	Ibrahim et al. [24]
Normal	21.16%		80.7%	0%
Supraventricular tachycardia	–	2.0%	3.6%	–
AF	8.76%	15.8%	4.5%	12%
Atrial flutter	–	0.20%	0%	2%
ST changes	14.09%	–		22% - 20%(ST depression) - 2%(ST elevation)
T-wave depression	–	–	–	20%
Tachycardias	5.05%	4.2%	–	54%
Bradycardia	12.86%		–	20%
Ventricular fibrillation (VF)	–	–	–	2%
Atrioventricular block	–	2.2%	0.6%	2%
Q wave abnormality	6.39%	–	–	2%
Prolonged QT interval	–	–	–	2%
Left ventricular hypertrophy (LVH)	–	–	–	48%
Pauses	–	–	3.4%	-

### Anticoagulation management for ischaemic stroke-related ECG changes

First-line treatments for acute ischaemic strokes involve intravenous thrombolytics, such as alteplase and tenecteplase, aimed to reperfuse ischaemic tissue [36]. Early administration is advocated for improved outcomes, despite the potential side effects of angioedema and intracerebral haemorrhage, necessitating the implementation of coagulopathy reversal protocols [36]. With ECG monitoring recommended in the initial 24 h post-ischaemic stroke, detected cardiac arrhythmias dictate the treatment pathway [37]. In cases of atrial fibrillation, oral anticoagulation should be initiated within 4—14 days following neurological symptom onset, utilising agents like warfarin or direct oral anticoagulant drugs (DOACs) such as dabigatran, edoxaban, rivaroxaban, and apixaban [38]. Still, these drugs pose bleeding risks [38]. Thus, UK guidelines recommend deferring oral anticoagulation for at least 14 days in patients with atrial fibrillation following a disabling ischaemic stroke, though in non-disabling strokes, earlier administration may be considered [39]. The timing for DOAC administration following atrial fibrillation identification remains uncertain but the treatment has proven effective [39].

### ECG-related mortality

Diverse ECG changes show associations with different stroke types, allowing for mortality estimation. Acute ischaemic stroke patients are more prone to sudden cardiac death, often due to VT [20]. As cardiac changes pose a higher risk 24 h post-stroke, Sylvan et al. found 44% of post-stroke patients without evidence of previous cardiac abnormalities to have arrhythmias on ECG monitoring [40]. Additionally, Scheitz et al. concluded that between 24 to 72 h is the peak time for acute cardiac dysfunction post-stroke and can persist up to day 14 [41]. Thus, it can be implied that ECG monitoring in post-stroke patients can assist in the early detection of cardiac-related complications [20]. Atrial fibrillation, the most common tachyarrhythmia post-ischaemic stroke, increases mortality risk due to its mechanisms involving blood stasis and atrial enlargement, raising the likelihood of systemic thromboembolism [15]. Other tachyarrhythmias, such as atrial tachycardia, exacerbate cerebral damage during or after a stroke, particularly in patients with cardiac disease [15]. While no ischaemic ECG changes predict excellent recovery, transient ischaemic ECG changes predict significant recovery, especially in moderate and larger infarcts [19]. However, persistent ischaemic ECG changes are more common in severe, large infarcts but cannot predict recovery likelihood [19]. It is essential to note that cerebral ischaemic occurrences are more likely in older patients, with over 65 year olds constituting 76.1% of all stroke cases in the UK in 2023/24, and overall, 86.6% were ischaemic [42]. Age-related physiological changes and ECG alterations increase arrhythmia likelihood, potentially influencing the study's findings [19, 43]. Arrhythmias have been linked to prolonged hospital stays post-ischaemic stroke [43]. Abnormal ECGs heighten the risk of heart disease, heart failure, coronary artery disease, and correlate with increased NIHSS and extended hospital stay [22, 43]. Tachycardia on admission increases mortality risk, but treatment has been shown to mitigate this risk [22].

As the intricate relationships between ECG changes and post-ischaemic stroke complications emerge from the aforementioned studies, the significance of cardiac monitoring in these patients becomes increasingly apparent.

## Left ventricular ejection fraction (LVEF) and ischaemic stroke

The co-occurrence of ischaemic stroke and low LVEF has been observed, and there appears to be a reciprocal relationship between the two, it is a more recognised predictor of stroke outcomes than other cardiac parameters like left ventricular stroke volume which are more recently investigated [44, 45]. This prompts the question: what does LVEF predict in stroke outcomes? Baker et al. reported that approximately 5% of ischaemic stroke patients exhibit a poor LVEF (< 40%) despite having sinus rhythm [8]. Demographically, stroke patients with both LVEF and sinus rhythm are more likely to be women and older (mean age 79.4 vs. 74.3) than those without these characteristics [46]. Similar to stroke-related ECG changes, different stroke-affected areas yield varied LVEF results. Surprisingly, the aetiology of ischaemic stroke has no association with LVEF. Ramasamy et al. compared ischaemic stroke patients with embolic stroke and other ischaemic stroke patients and found no significant difference in the LVEF in those patients [47]. Although no relationship between the cause of ischaemic stroke and LVEF was found, the location of stroke offers differences in LVEF [48]. In a study by Min et al. involving 415 ischaemic stroke patients, the middle cerebral artery (MCA) was the most affected artery in stroke. Specifically left MCA infarcts with insular involvement displayed the lowest LVEF average of 45%, compared to the highest LVEF average of 58% in MCA infarcts without insular involvement [48].

### LVEF and stroke

Transiently low LVEF in ischaemic stroke may be attributed to stunned myocardium, a condition where cardiac dysfunction is typically reversible within days to weeks, with most patients experiencing an improvement in LVEF within four weeks [49]. This phenomenon occurs independently of coronary artery blockage, as evidenced by angiography findings in these specific patient subpopulations across both stroke subtypes [50–52]. Echocardiography further supports this, revealing apical sparing in the context of systolic dysfunction [53, 54]. The cause is thought to be secondary to central nervous system injury inducing autonomic dysfunction, leading to increased sympathetic output and catecholamines, resulting in coronary artery vasospasm and an oxygen demand exceeding supply [55]. As mentioned previously, lower LVEF is more associated with a left-sided MCA strokes affecting the insular cortex, a structure contributing to autonomic heart stimulation [48]. However, stunned myocardium is not solely attributed to insular involvement, as a separate study by Murthy et al. found that less than 40% of patients had insular involvement [49].

Furthermore, the insular is an important area in the brain for receiving feedback signals from the heart such as the baroreceptors and myocardial visceral information, to ensure appropriate cardiovascular autonomic control [56]. Winder et al. found that lesions in the right posterior insula can affect sympatho-inhibitor control, which increases catecholamine-related myocardial toxicity, leading to reduced LVEF post-stroke [57]. Other areas of the brain with close links to the insular, such as the amygdala and hippocampus, have also been associated with sympathetic control of the cardiovascular system and can explain the cause of reduced LVEF post-ischaemic stroke [56].

### LVEF as predictor for stroke outcomes

In addition to the association between LVEF with sinus rhythm and stroke, LVEF may be used to predict stroke outcomes. Clinically, LVEF is used to categorise heart failure (HF) into those with preserved (HFpEF) or reduced ejection fraction (HFrEF) [58]. Correlatingly, stroke patients with heart failure (HF) have shown reduced short-term (80.6% vs. 88.6%) and long-term (19.4% vs. 44.1%) survival rates compared to stroke patients without HF. This suggests a potential link between stroke outcomes and LVEF, which is concurrently associated with higher stroke mortality and worse clinical presentations post-stroke [44]. Conversely, stroke patients with higher LVEF have better outcomes [38]. Rojek et al. recognised higher LVEF in patients with significant post-stroke improvements compared to those without (54.3% vs. 49.9%) in the short term [59]. Four multivariable models conducted in the study showed almost a threefold improvement in stroke patients with preserved LVEF. It was also noted that lower LVEF ischaemic stroke patients are more adversely affected by arterial stiffness, a known strong predictor for future cardiovascular events, than normal LVEF ischaemic stroke patients [59, 60]. However, as coronary artery disease (CAD) affects post-stroke prognosis and some participants refused further CAD testing, caution is needed in interpreting the study's findings [59]. Although to support the above, a longitudinal study by Lee et al. found that lower LVEF was associated with a significantly higher rate of all-cause mortality in acute ischaemic patients without AF or coronary heart disease [61].

Equally, Ois et al. supported Rojek's results and concluded that patients with ischaemic stroke and lower LVEF are more likely to experience poor stroke outcomes than those with higher LVEF, with odds ratios of 3.0 and 2.5, respectively [24]. Limitingly, some ECGs utilised were conducted prior to the stroke incidents, which does not completely rule out previous CAD before the stroke, potentially contributing to a worse prognosis. Li et al. found a significantly worse stroke outcome in ischaemic stroke patients with lower LVEF (60%) than those with higher LVEF (62%), albeit with marginal differences [62]. Other findings included a potential relationship between left ventricular systolic dysfunction, defined as LVEF < 40%, and poor ischaemic stroke outcomes [62]. Interestingly, Li et al. also suggested that LVEF may be used as a predictor of AF in stroke patients based on the relationship found between HF and AF [62].

As an alternative approach to measuring the relationship between LVEF and ischaemic stroke, Adams et al. utilised the NIHSS to determine the effectiveness of LVEF in predicting ischaemic stroke outcomes [63]. The NIHSS score is clinically considered a good predictor for outcomes of ischaemic stroke [63]. A score of more than or equal to 16 indicates a high possibility of death or severe disability post-stroke, while a score of less than or equal to 6 suggests a good recovery [63]. Based on this guide, Adams et al. found that patients with NIHSS scores ≥ 6 had lower LVEF compared to those with NIHSS scores < 6 (61.3% vs. 68.6%, respectively) [63]. Therefore, it can be interpreted that lower LVEF may indicate poorer ischaemic stroke outcomes, either directly or indirectly.

Contrary to the above, Mathias et al. did not find LVEF to be a significant predictor of ischaemic stroke outcomes. However, this study was limited to a small sample size, consisting only of patients with moderate or severely low LVEF [64].

Overall, patients with a reduced LVEF (≤ 60%) and acute ischaemic stroke had a lower survival rate one year post-stroke than patients with high LVEF (OR 1.29, 95% CI 1.06–1.58). Although LVEF of 50–60% is considered normal, Wei et al. still suggested poorer outcomes for those patients also [65]. They found a significantly increased risk of recurrent stroke one year after the event (OR 1.14, 95% CI 0.99–1.32) in patients with LVEF of 56–60% [65]. Additionally, no other LVEF ranges, either lower or higher, were found to be significant. It is noteworthy that this study had a small sample of patients with reduced LVEF, and this may explain the lack of significant results found in patients with LVEF < 56%.

Based on the evidence presented, LVEF is a factor contributing to the outcomes of stroke patients. However, contradicting evidence exists regarding its efficacy, and no studies have been conducted to suggest its superiority against existing predictors such as the NIHSS score. There is also limited evidence on the direct associations of stroke size, haemorrhagic transformation of ischaemic stroke, and increased intracranial tension as a complication of ischaemic stroke with LVEF.

### Diagnosis and management of low LVEF and sinus rhythm in stroke

Currently, NICE guidelines recommend an ECG after an ischaemic stroke, primarily to identify a cardiac cause of ischaemic stroke, usually AF, rather than to assess any injurious effect of the stroke [66]. This is supported by the guidelines from the World Stroke Organization in patients with a clinical history of, or evident with heart disease [67]. Other recommendations from the European Stroke Organisation included continuous ECG monitoring for up to 30 days in patients who have had an embolic stroke of unknown origin [68]. However, brief episodes of AF during the monitoring can indicate a reduction in future recurrent strokes if treatments are applied adequately. Alternative methods to detect myocardial dysfunction post-stroke could include cardiac biomarkers. Cardiac troponin T and brain natriuretic peptide (BNP) have previously been linked to low LVEF post-stroke [48], and cardiac troponin I has previously been used to show cardiac dysfunction in patients who have suffered from subarachnoid haemorrhage [69]. To definitively visualise dyskinetic myocardium and calculate ejection fraction, a common method is echocardiography. Another imaging modality that has shown success in identifying ischaemic stroke patients with low LVEF is cardiovascular MRI and cine real-time imaging, which possesses advantages like not being reliant on an acoustic window and having reduced assessment time, among others [70].

Managing low LVEF post-stroke does not have a homogenous approach. A multicentre study in the USA found that, overall, patients with lower LVEF were more likely to be anticoagulated on discharge, although the proportion of patients anticoagulated varied between sites [8]. Anticoagulation in post-stroke patients is used for the prevention of aetiologies of stroke including AF, venous or cardiac thrombus formation, patients with mechanical heart valves, and cancer-related stroke. However, the direct benefits of anticoagulation in certain aetiologies of stroke are unknown, such as cancer-related hypercoagulability, and much debate is present around when anticoagulation should be started and how long it should be used. Although stroke risk increases with decreasing LVEF in sinus rhythm, results regarding anticoagulation to prevent future stroke risk have differed depending on the agent used. Vitamin K antagonists such as Warfarin were not shown to be beneficial in reducing stroke risk and carry the risk of significant bleeding [71]. Data for and against the benefit of Rivaroxaban in this clinical scenario exist. The COMPASS trial observed decreased future risk of stroke, although with less efficacy in those with lower LVEF [71, 72]. In contrast, the COMMANDER-HF trial did not find a significant decrease in future stroke in those with reduced LVEF [73].

## Stroke-heart syndrome

Stroke-heart syndrome is a neurogenic condition characterised by cardiac complications following a stroke during the first 30 days post-stroke [74]. The 5 main cardiovascular complications as a part of stroke-heart syndrome are acute coronary syndrome, left ventricular dysfunction, myocardial injury, electrical abnormalities, and sudden neurological death. Although the full pathophysiology remains incompletely understood, ongoing research suggests a complex interplay involving ischaemic stroke-lesion characteristics, such as size and severity, various local cerebral and systemic mediators, and downstream cardiac mechanisms like impaired coronary microcirculation, macrophage dysfunction, and cardiomyocyte injury [41]. Interestingly, this neurocardiogenic injury has also been observed in other acute neurological disorders, including seizures, intracranial haemorrhage, and traumatic brain injuries [75]. Despite our evolving understanding, this syndrome has been linked to an increased risk of VT, VF, and HFrEF, contributing to a twofold rise in post-stroke mortality risk [76].

### Presentation

Stroke-heart syndrome lacks a specific presentation, manifesting in various forms such as heart failure, arrhythmias, systolic and diastolic dysfunction, ECG abnormalities, and/or cardiac arrest [77]. Cardiac complications rank as the second most common cause of mortality in the acute phase of post-ischaemic stroke, surpassed only by direct neurologic complications [13]. Furthermore, the presence of cardiac complications negatively impacts long-term stroke outcomes, emphasising the importance of understanding the diagnosis and management of stroke-heart syndrome [13]. However, sometimes medical history cannot be established and no clinically relevant differentiation criteria have been found for novo or pre-existing cardiac conditions when researching for this literature review. However, Mochmann et al. have established that even though acute ischaemic stroke and acute coronary syndrome both have similarly raised levels of the cardiac troponin cTn [78], acute ischaemic stroke patients were less likely to be found with “coronary culprit lesions” and half of these patients had no evidence of coronary artery disease [78]. This can be implied that acute ischaemic stroke patients often present with minimal pre-existing cardiovascular diseases. Therefore, any changes present in ECG cardiac diseases post-stroke are much more likely to be transient than chronic.

### Epidemiology

Numerous studies have delved into the epidemiology of stroke-heart syndrome, revealing a substantial risk of developing cardiac complications post-stroke. Gunnoo et al.'s meta-analysis, incorporating 17 studies, reported asymptomatic coronary artery disease in an average of 52% of patients with acute ischaemic stroke [79]. Additionally, even patients without a history of cardiac dysfunction faced a 3% risk of myocardial infarction one year after a stroke [79]. Burns et al.'s population-based study investigating myocardial infarction incidence in TIA patients found an average annual incidence of 0.95%, double that of the general population, particularly affecting those under 60 [80].

### Risk factors

Markers of cardiac function, including carotid artery stenosis and left ventricular ejection fraction percentage, were significantly associated with stroke-heart syndrome in Lian et al.'s study [81]. Among other factors, such as age, sex, activated partial thromboplastin time (APTT), prothrombin time (PT), D-dimer, neutrophil count, and the NIHSS [81]. In the study by Powers et al., myocardial markers were identified as potential diagnostic indicators for stroke-heart syndrome upon admission [82]. It is also recommended for regular hs-cTn and prolonged ECG monitoring for early detection of possible severe cardiovascular complications, as cTns are known biomarkers for injury to the myocardium [41].

### Investigations

In the USA, cardiac troponin is recommended as the biomarker of choice by the American Stroke Association when investigating suspected stroke-heart syndrome [83]. Approximately 20% of patients exhibit elevated cardiac troponin levels following an ischaemic stroke [83]. Fan et al.'s meta-analysis revealed that elevated cardiac troponin levels were associated with a 2.54 times higher risk of in-hospital mortality and an 89% overall mortality risk [84]. However, in the UK, the National Institute for Care Excellence (NICE) and the Scottish Intercollegiate Guidelines Network (SIGN) do not recommend using troponin levels to investigate stroke-heart syndrome, possibly due to an incomplete understanding of the exact aetiology of elevated cardiac troponin in stroke patients [84]. Further research in this area is essential for developing more effective guidelines for diagnosing stroke-heart syndrome.

Lian et al. developed PANSCAN, a risk prediction scale for identifying high-risk patients for stroke-heart syndrome, based on a logistic regression model with a sensitivity of 0.935 and specificity of 0.720 [81]. Scores ranging from 0–10 were assigned, with those scoring 3 or higher identified as high-risk [81] Table 2.

**Table 2:** PANSCAN risk prediction scale for stroke-heart syndrome scale developed Lian et al. [81]

Risk Factor	PANSCAN Score
PT	12-14 s = 0
	Otherwise = 1
APTT	30-45 s = 0
	Otherwise 1
Neutrophils	50–70% = 0
	Otherwise = 1
Sex	Female = 1
Carotid artery stenosis	Normal or mild = 0
	Moderate to severe = 1
Age	≥ 65 years = 1
NIHSS score	1 to 4 = 2
	≥ 5 = 3

While consensus exists regarding increased age as a risk factor for stroke-heart syndrome, Lian et al. assigned a low score of 1 for higher age, diverging from this common understanding [81]. In contrast, Burns et al. found that patients below 60 were at a higher risk of developing cardiac complications post-stroke compared to older individuals [80]. Therefore, relying solely on higher age as a risk factor might not be as effective in predicting post-stroke outcomes.

### Management

Limited literature exists on the most appropriate treatment for stroke-heart syndrome. Beta blockers are generally recommended to prevent cardiac remodelling and hyperactivation of the sympathetic nervous system following a myocardial infarction; however, evidence supporting their benefits after an ischaemic stroke is inconclusive [77].

A systematic review by Romiti et al. suggested that following the ABC (Atrial fibrillation Better Care) pathway, known to reduce all-cause death, death due to cardiovascular causes, stroke, and major bleeding in patients with atrial fibrillation, might be beneficial for stroke-heart syndrome [85, 86]. The ABC pathway is a three-part process, aimed to improve and simplify stroke prevention in patients with AF. It involves prescribing appropriate antithrombotic agents, improving a patient's functional and psychological status, and minimising cardiovascular risk factors through the effective management of comorbidities [87, 88]. Lifestyle changes, education, and counselling in primary care settings are recommended as part of the treatment plan [88]. Specifically, the pathway utilises the Birmingham 3-step method to identify patients with AF and at low risk of stroke who have a CHA2DS2-VASc score of 0, offer stroke prevention, and consider a suitable oral anticoagulant such as a vitamin K antagonist or novo oral anticoagulants [86, 89]. Additionally, recent research by Tsang et al. has found adherence to the ABC pathway to have significantly reduced overall mortality post-stroke (HR 0.72, 95% CI 0.62–0.85), including cardiovascular deaths (SHR 0.64, 95% CI 0.45–0.90) in a large cohort [90]. These results suggest promising preventative measures for stroke-heart syndrome in all post-stroke patients. However, no distinguishing clinical research regarding the ABC pathway and stroke-heart syndrome has been conducted.

In summary, the pathophysiology of stroke-heart syndrome remains incompletely understood, but it is believed to result from the interaction between ischaemic lesions, local and systemic mediators, and downstream cardiac mechanisms. The discussed literature highlights the prevalence of this condition among stroke patients, emphasising the need for further research to ensure its appropriate management. Current clinical practices lack consensus on the best approach to managing and treating stroke-heart syndrome. Ongoing investigations include testing for biomarkers like cardiac troponin, and a risk prediction scale has been developed to assist in the diagnosis of this syndrome. The ABC approach, involving antithrombotic agents, improving functional and psychological status, and minimising cardiovascular risk factors through comorbidity management, has been suggested as a potential treatment plan for stroke-heart syndrome.

## Conclusion

This literature review explored cardiac changes post-ischaemic stroke, the relationship of these changes with post-stroke outcomes, and their abilities to predict outcomes. We discussed the stroke related cardiac changes involving ECG and LVEF and reviewed the current development into the stroke-heart syndrome. In contrast, these ECG changes or LVEF levels can be causes, effects, or associations. Therefore, statistical analysis is critical to establish if these relationships are trustworthy as a predictors for ischaemic stroke outcomes or complications.

Specifically, ECG monitoring is vital between 24–48 h after stroke as the optimal time to monitor for arrhythmias or ischaemic changes and detect potential cardiac damage. These arrhythmias are often associated with the location and size of the ischaemic stroke and outcomes. ECG abnormalities were associated with prolonged hospital stays and worse outcomes. Overall, arrhythmia post-ischaemic stroke correlates with higher mortality and severe complications including VT, AF-related thromboembolism, and sudden cardiac death.

Similarly, LVEF also has an equally strong relationship with post-stroke complications. Lower LVEF increases both short-term and long-term stroke mortality and heighten the risk of future cardiovascular events. Contrastingly, higher LVEF is associated with greater recovery post-ischaemic stroke with much-improved outcomes. Currently, there is minimal guidance on the management of patients with lower LVEF post-ischaemic stroke. However, rivaroxaban is promising and reduces the risk of future stroke and haemorrhages.

Lastly, stroke-heart syndrome may have an important and deadly role in post-stroke patients. It includes severe cardiac abnormalities of acute coronary syndrome, left ventricular dysfunction, myocardial injury, arrhythmia, and sudden neurological death. Stroke-heart syndrome has shown poor long-term outcomes in ischaemic stroke patients. Additionally, pre-existing cardiac changes further elevate the risk of stroke-heart syndrome. Although there are no current guidelines on the identification and management of stroke-heart syndrome, the PANSCAN tool and the ABC pathway are significant in the identification and mitigation of risks and mortality in stroke-heart syndrome.

**Fig. 1:**
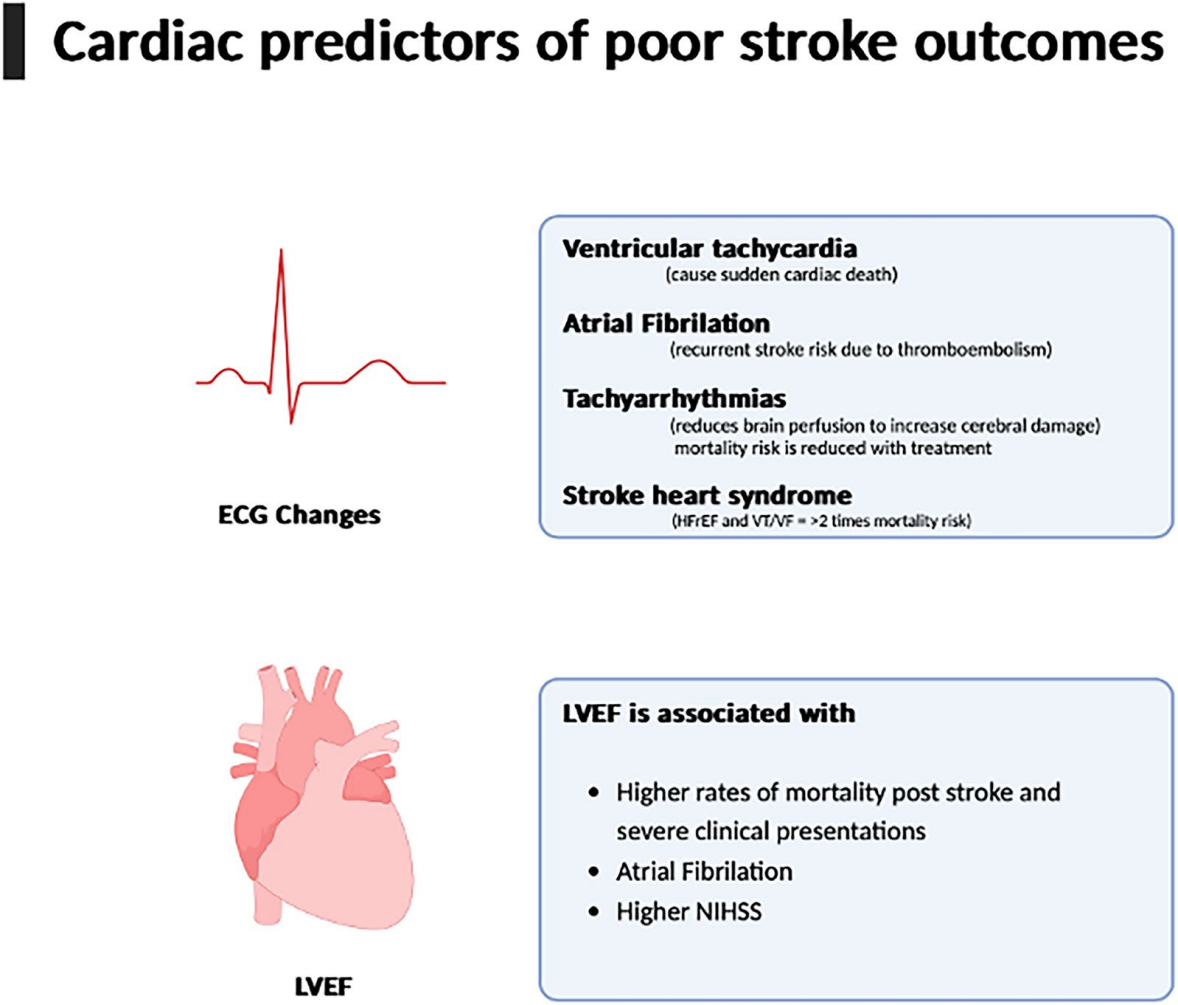
Cardiac predictors of poor stroke outcomes. ECG, electrocardiogram; HFrEF, hear failure with reduced ejection fraction; LVEF, left ventricular ejection fraction

To conclude, we have found valuable information on how ECG and LVEF can be used to recognise poor outcomes, and potential management options for prevention (Fig. 1). We also discussed the importance of stroke-heart syndrome and the current resources available. We want to reinforce the critical need for ECG and LVEF monitoring for the prediction of mortality and morbidity, as well as  the recognition of stroke.

### 5.1 Further Research

Current evidence has shown a correlation between ECG changes and stroke outcomes, but more patient data are needed to prove the benefits of utilising ECG changes in the clinic, to estimate ischaemic stroke outcomes. Some research has been found linking lowered LVEF with sinus rhythm, with ischaemic stroke. However, due to similarities in pathophysiology, it is unclear which caused the other. Like above, more concrete evidence is needed if this was to inform clinical practice. Additionally, further research needs to be done to ascertain the most effective way to monitor cardiac complications post-stroke and identify whether these are a cause or consequence of ischaemic stroke. Even though stroke-heart syndrome is rare post-ischaemic stroke, there are gaps of research in the current literature with regard to stroke-heart syndrome and its management guidelines. Therefore, further understanding of stroke-heart syndrome pathophysiology may aid in the development of management guidelines.

## Data Availability

Not applicable

## Abbreviations

AF: Atrial fibrillation
APTT: Activated partial thromboplastin time
BNP: Brain natriuretic peptide
CAD: Coronary artery disease
DALYs: Disability-adjusted life years
DOACs: Direct oral anticoagulants
ECG: Electrocardiogram
GBD: Global Burden of Disease
HF: Heart failure
Here: Heart failure with reduced ejection fraction
LVEF: Left ventricular ejection fraction
LVH: Left ventricular hypertrophy
MCA: Middle cerebral artery
NICE: National Institute of Care Excellence
NIHSS: National Institutes of Health Stroke Scale
PT: Prothrombin time
SIGN: Scottish Intercollegiate Guidelines Network
TIA: Transient ischaemic attack
VF: Ventricular fibrillation
VT: Ventricular tachycardia
